# A qualitative study of the perceptions and experiences of Pre-Registration House Officers on teamwork and support

**DOI:** 10.1186/1472-6920-5-10

**Published:** 2005-03-09

**Authors:** Heidi Lempp, Mac Cochrane, John Rees

**Affiliations:** 1 Guy's, King's and St. Thomas' School of Medicine, King's College, London Sherman Education Centre, 4^th ^Floor, Guy's Hospital, London SE1 9RT, UK; 2 Academic Rheumatology, Guy's, King's and St. Thomas' School of Medicine, King's College London, Weston Education Centre, Cutcombe Road, London SE5 9RJ, UK

## Abstract

**Background:**

Following the implementation of a new final Year 5 curriculum in one medical school we carried out a study to explore the experience of the transition from final student year to Pre-Registration House Officer (PRHO). This study looks at the experiences of two successive cohorts of PRHOs in relation to team work, support and shared responsibility in their transition from final year students to qualified doctors. The involvement of PRHOs in teams is likely to change in the development of Foundation programmes.

**Methods:**

A qualitative study with semi-structured interviews with 33 PRHOs, stratified by gender, ethnicity and maturity, from two study cohorts, qualifying in 2001 and 2002, from one medical school in the UK, in their first three months following medical graduation.

**Results:**

Most PRHOs reported positive experiences for their inclusion as a full member of their first ward teams. This contributed to their increasing confidence and competence in this early period of career transition. However, a number of organisational barriers were identified, e.g. incomplete teams, shift work, which produced problems in their integration for one third of newly qualified doctors.

**Conclusion:**

Recently introduced policies, intended to improve the working lives of newly qualified doctors have produced both benefits and unintended adverse impacts on PRHOs. The changes of the new PRHO Foundation programme will have further impact. Foundation doctors may need to relate to wider teams with more interaction and less protection. Such changes will need to be managed carefully to protect the PRHO at a vulnerable time.

## Background

There is increasing emphasis on multi-disciplinary teams in modern clinical care [1-3]. Medical schools have attempted to embrace this through interdisciplinary learning [1,4-6] and stressing relevant attitudinal, ethical and behavioural issues [7-9] in relation to teamwork.

Concern has been expressed about whether such teamwork skills can be effectively practised by junior doctors during the PRHO year while they cope with multiple professional, personal, physical and emotional demands [10-12]. The General Medical Council document, *The New Doctor *[13] stated that one of the professional learning outcomes of the pre-registration year is 'to work in a team and to take collective responsibility for patient care'. Several authors [14-17] have recognised difficulties in delivery of these goals although most PRHOs appear to acquire the ability to work in a team by the time of qualification [18,19]. More recent policy changes, such as The New Deal [20] might reduce the contribution of PRHOs to teamwork and decrease job satisfaction [21].

We wanted to explore how well our undergraduate curriculum, particularly the final year with 16 weeks shadowing in a student house officer role, prepared PRHOs for their new posts. In this study issues emerged about the stability of ward teams and the full integration of PRHOs in to teams. These findings may have relevance to the development of Foundation programmes.

## Methods

Two study cohorts of 33 PRHOs were selected by convenience (16 participants from year 2000/2001) and quota sampling (17 participants from year 2001/2002), stratified by gender, ethnicity and maturity, using the respective final year student population of the Medical School as the sampling frame (see Tables 1 &2). Written, informed consent was obtained from each participant. Semi-structured 1:1 interviews (see Additional file 1) were carried out within the first three months of their PRHO posts. Students' accounts were evaluated against the relevant professional competencies set out in *The New Doctor *[13] during a comprehensive evaluation of the newly developed final year in the medical school. This paper focuses on issues related to the role of the doctor within the health service, particularly 'the ability to work in a team' and 'accept principles of collective responsibility' [12]. All interviews were tape-recorded and transcribed in full. Two of the authors and an independent researcher read through the transcripts and identified a list of emerging themes. These lists of themes were then cross referenced and amended by the readers to reach agreement for the analytical framework of content analysis [22]. Throughout the data analysis the occurrence of key findings are noted by using simple counts. Combining qualitative and quantitative data allowed the authors to unpack, confirm and emphasise some similarities or differences between the different study cohorts. This approach assists in the generalisability of the findings [22] and offers evidence more convincingly (but not in a statistical significant sense) than relying on anecdotal accounts alone. This method can also contribute to the validation and credibility of qualitative research [22-24]. There was one key question in reference to team work but relevant information from all parts of the interview was used in the analysis (see Appendix 1).

**Table 1:** Socio-demographic data from 16 PRHO cohort 2000/2001

**Gender**	11 Females, 5 Males
**Age**	24 years (mean)
**Ethnicity (self-described)**	5 Indian, 1 Gujarati, 1 Irish-Indian, 1 English-Chinese, 1 Sri-Lankan, 7 Caucasian
**Class**	13 middle class, 3 working class
**Family status**	15 single, 1 married
**Country of birth**	15 UK, 1 outside Europe
**Religion**	5 Christian, 3 Hindu, 5 none, 3 not stated
**Entry to Medical School**	11 after school, 2 after a gap year, 3 mature (3/16 obtained intercalated BA/BSc during their time at Medical School)

**Table 2:** Socio-demographic data from 17 PRHO cohort 2001/2002

**Gender**	9 Females, 8 Males
**Age**	24 years (mean)
**Ethnicity (self-described)**	1 Black-African, 1 East Asian, 7 South Asian, 8 Caucasian
**Class**	14 middle class, 3 working class
**Family status**	16 single, 1 married
**Country of birth**	14 UK, 3 outside Europe
**Religion**	5 Christian, 3 Hindu, 1 Islam, 6 none, 2 not stated
**Entry to Medical School**	12 after school, 1 after a gap year, 1 after A level retake, 3 mature (10/17 obtained intercalated BA/BSc during their time at Medical School)

## Results

The 33 PRHOs worked in four acute hospital trusts and seven District General Hospitals (DGHs) in England, 16 in surgical and 17 in medical wards. They commented frequently on the importance of their clinical teams. Findings from PRHOs' accounts are reported around 3 key themes: supportive environment, educational environment and organisational changes.

Overall the transition from being an 'outsider' to a professional 'insider' was a welcome change for all PRHOs, including involvement in the team. Comments on finding a role were common:

'When I was a medical students in the 5^th ^year I was very much aware that that I didn't have a role, I felt I was sort of very superfluous to the process... 'Now I have, a definite, a clear cut role, and I know what I should be doing, and what I shouldn't be doing, and I have a continuity I have my patients and I have a team, which I have a good relationship with'.

'In the previous years we haven't been actually part of a team. We've been assigned a team but you're not working with a team....... you're not part of that patient's everyday care'.

'It's so different being a house officer than being a medical student – you're a team, like, straightaway. The first day, that's it! That's a very big difference.'

### 1) Supportive environment

Most PRHOs (27/33) described their clinical teams in positive terms (see Table 3). Even so they also identified a number of specific characteristics, which had a profound influence on their first weeks working as qualified doctors.

**Table 3:** Descriptions by 27/33 PRHOs about their medical and surgical ward teams

'The team is fine'; 'the team is working brilliantly, we gelled really quickly'; 'definitely working well' (x2); 'working well together sometimes not so well'; 'definitely I feel part of the team' (x5); 'the team is great now, but not in the first two weeks'; 'felt part of the team quickly, very quickly'; ' feel part of the team, very much so'(x2); 'it's a really nice team'(x2); 'there is a good team spirit'; 'we work as a team' (x2); 'I really like my team, they are very supportive'; our team functions very well, everybody is friendly, chats and makes jokes'; 'there has been a welcome surprise, such as the team, it has been very supportive'; 'and I got a really nice team'; 'I work well with the team and felt accepted'; 'definitely I am part of the team, you're a sort of team player really'; 'oh yeah I get on really well with them'.

#### Constructive feedback from senior colleagues

PRHOs reporting support by senior medical staff (24/33) felt that they integrated with their clinical teams early and well, especially in emergency situations. Acute or life threatening events affected them emotionally, usually with little time for debriefing. Without senior support PRHOs often felt out of their depth. When they had dealt independently with a complex clinical situation, constructive feedback from senior staff added to their positive experiences. Conversely PRHOs who received no or little supervision or response from senior staff recorded these events as a negative impact on their confidence. The latter situations were more common in surgery, where PRHOs reported that senior staff was less accessible.

'I would have quite liked an SHO [Senior House Officer] or somebody to tell me what I should be doing in that situation, or whether I should be phoning the GP or writing or speaking to him, or who actually should be talking to her [patient] and what actually telling them [the couple] ...ahm...so I basically did it all myself.'

'...getting called to see a man that had collapsed on his way back from the loo, and had no blood pressure and was basically dying. And I thought: Ahhh! But I just called the SHO and said: "Can you come and help me?" And I couldn't get access in him [patient] at all, and it was just a bit scary. When the SHO arrived we sorted him out and he was then fine after that, although he died a few days later'.

#### Sharing responsibilities and tasks

A sense of collective responsibility with other team members was positively expressed by most PRHOs (26/33). The predominant ethos was one of sharing the workload between medical and nursing colleagues in the team (see Table 4).

**Table 4:** Phrases used by 26/33 PRHOs about sharing work load and responsibilities

'jointly' (x 2), 'share' (x5) 'do the jobs' (x8), 'help' (x3), 'we do the same jobs', 'split the jobs', work generically as a team', 'divide jobs up', 'catch up with all the jobs', 'we have jobs to do between the house officers and all the colleagues', 'do all the work together', 'work together closely', 'plan our jobs', 'carrying out the jobs together', 'manage jobs together on the wards', 'taking care of referrals', 'do jobs as we go along' [during the ward round], 'we finish of jobs', we sort out our jobs'.

*some used more than one phrase

PRHOs reported that sharing jobs was not always straightforward, particularly if individual team members did not work well together (7/33), or if teams were incomplete due to staff shortage and/or locum cover (8/33).

'I mean it's [work] so erratic and our team is so disjointed! I don't really know half the patients and I don't...I don't even know if half the jobs are getting done. I mean mine all get done but I don't know whether the other half's, the other House Officers are thinking I'm doing theirs or they're doing mine, or the SHO's doing whatever'.

#### Interprofessional working

28/33 PRHOs reported that the relationship with nursing staff changed with qualification. Former inter-professional tensions seemed due to the perceived inconvenience that medical students caused to nurses. Prior to qualification students had relied on nurses in hospital and community for help with 'log book' skills, such as catheterisation and injections, and they appreciated their patience and enthusiasm.

'Before as a medical student you were a piece of furniture according to nurses....but now, obviously you are the first-line if they want anything doing, I think in that respect there's a lot more regard....and appreciation for what you do'.

However, a number of inter-professional conflicts were identified such as adjustment to professional roles (x 15), discordant communication (x9), unclear what nurses are willing or (un)able to do (x10) and others.

#### Relationship with PRHOs in the same team

8/33 commented positively on working with other PRHOs from their final year group in the same team, making sharing the workload and responsibility easier due to closer personal relationships. However, 2/33 identified this as impacting on their teamwork and patient care when PRHOs did not get on well together and 4/33 found competition to learn additional clinical skills.

'There always has been camaraderie with other PRHOs, but at least I get to spend time around now as well, so ahm...you know it is great, when you literally you say, will you do this, I will do that, and we meet up at the end [of the day]....that is always really nice'.

'There's a mutual understanding there (amongst the PRHOs in the team], and we...amazingly, we are ever so professional on the job. I mean on the team, we're very good friends – we go out for dinner, all sorts – but when we're on the job, it's ever so professional'.

### 2) Educational environment

PRHOs sought out educational opportunities to achieve a sense of progression after qualification within their clinical teams. Learning consisted of performing new practical skills, as well as observation, to acquire inter-personal and professional competencies. Continuity in their relationship with patients was perceived as important as team members, rather than temporary bystanders as students.

#### Observing delicate situations

Most PRHOs (23/33) had witnessed delicate clinical or ethical situations, which required advanced communication skills between team members, patients and their families, e.g. breaking bad news, communicating with uncooperative patients. The interviewees had a variety of opportunities to observe how senior medical and nursing staff within their teams managed such situations sensitively and professionally.

'....it is quite interesting, watching your seniors how they deal with difficult patients: patients who are too demanding, or patients who, you know, are rude or angry, or breaking bad news, I try to sort of sit in on all that, so I can see how they [seniors] do it....'

' ... when family gets involved, it gets awfully sad [resuscitation status]. I had a patient who died; I got to know the family relatively well – two sons – and they divulged that their mother had died, and...all sorts of complex family dynamics going on there. They were quite clearly worried about their father. And then there's only so much input the house officer can do – essentially, the seniors make the decisions...'

PRHOs were acutely aware that consultants were ultimately in charge of final decisions in ethical dilemmas. 6/33 PRHOs reported that they were given tasks which they felt were inappropriate, such as asking patients for consent to undergo an operation or investigation which they had never seen themselves.

#### Learning new clinical skills from seniors

7/33 PRHOs were able to extend their practical skills, such as lumbar punctures or insertion of chest drains during their first house jobs. However, these opportunities were reduced where ward teams were incomplete and depended on the willingness and clinical competence of senior staff in their teams. Such limitations sometimes caused frustration. The message around unstable teams was often close to:

'Our team's so disjointed at the moment. The SHO that we've got is constant. But, she does 'on-calls' for other teams as well so we don't see her that often. Our Registrars change every day because they're all locums. So on some days we don't have one and some days we do ...I don't feel....I'm not learning the things I thought I'd learn.'

'There's only one regular member of the team and that's the staff grade, and so the consultant is a locum. He's been here a couple of months, or just before we started, basically he was here. The SHO is a locum SHO, who has now finished her rotation, and I've just started so there was not much stability in the team to begin with.'

### 3) Organisational changes

Organisational changes, such as new work patterns, and inadequate staffing levels caused frustration and anxiety affecting the PRHOs emotionally and their team work.

#### Continuity of relationships with patients

Most PRHOs (25/33) described continuity of patient care in positive terms. They welcomed the fact that they could approach patients without apologies because they had a defined role and responsibility. 11/33 PRHOs reported fragmentation in their relationships with patients which they related to shift work and/or staff shortages.

'...the continuity is great, you know, walking on the ward, and sort of saying "hi" to people and you know they know you, and getting on with them and kind of trying to do your best for them.'

'....there's no continuity of care: we start in the morning, we finish, and then someone else comes on – and they [consultant] don't know the person. And then the next day that patient either moved to somewhere else or they've gone home. So we're not following them [patients].'

#### New work patterns

One third of PRHOs in study cohort 2001/2002 confirmed that their hospital complied with new working hours while two thirds encountered difficulties.

The 2001/2002 group had a range of concerns: continuous long working hours, despite the implementation of new working patterns (12/17), loss of emergency experience at night (4/17), confusion from non-compliance or experimentation with new working hours (12/17), and unhappiness and ridicule by senior colleagues (5/17). These caused resentment and dissatisfaction amongst the majority of the cohort (12/17) more than the 2000/2001 group (5/16).

#### Staffing levels

In addition to the new working arrangements, there was a change from 2000/01 to 2001/02 in the availability and continuity of permanent staff members, doctors and nurses. This was reported only by the second PRHO cohort. One third of this group commented that team work was disrupted and undermined by incomplete teams.

'It's just working in a big hospital, and having an incomplete team was...it was just unfortunate that that was the situation when I joined...I didn't enjoy that and it upset me because, you know, it's taken me a long time to get through Medicine and I felt that that was bad.'

## Discussion

In a few areas such as changes in rotas and hours the interviewees commented on the views of more senior doctors. These views may have influenced their responses. In order to encourage open responses interviewees were guaranteed anonymity. Interviews were conducted mainly by one interviewer, who was not part of the medical school hierarchy or trusts and had established a good rapport with the PRHOs cohorts. The interviews in this study ranged over a number of areas. They explored the preparation of PRHOs through their undergraduate curriculum and the sources of stress in the transition to the PRHO role. PRHOs discussed their experience of the working environment and their relationships with colleagues in a number of areas and team work was addressed specifically in a direct question. The themes explored in this study were identified clearly by the three authors and the independent researcher on analysis of the transcripts.

The PRHOs were enthusiastic about the way their assumption of their professional role allowed integration into multi-disciplinary working with a clear change in the working relationships with nursing colleagues. This was an area that changed from their medical school experience despite efforts to give them clearly defined patient management roles during 16 weeks of experience as student house officers in hospital.

It is possible that interprofessional education and even clearer integration of student house officers may help to minimise the changes on transition in future. Clear evidence about the efficacy of interprofessional education, however, has not yet been established.

The supportive and educational environments facilitated by ward teams received a welcome positive reaction from the majority of newly qualified PRHOs in both study cohorts. The importance of adequate senior supervision in the early stages of the PRHO experience has been emphasised in previous studies [25]. Opportunities to observe senior staff in clinical situations were especially useful.

The New Deal [20] and the New Doctor [13] were intended to improve working conditions for PRHOs. It has been suggested that introduction of 'new deal' rotas may increase psychological morbidity and reduce job satisfaction for PRHOs [21], but that a well supervised working environment may counteract reductions in hours with high work intensity [26]. In this study difficulties with working patterns appeared to have increased between the two cohorts. Since this study was performed considerable further effort has gone in to the design of compliant and suitable rotas for doctors in training.

The problems were exacerbated by instability of the working teams which meant that supervision was not always adequate at these times when it needed to be most effective. Shortage of permanent staff in the ward teams was stressful and unsettling for one third of the PRHOs. These organisational difficulties affected five key areas of work: continuity of patient care, sharing responsibilities within the team, delayed integration in to the new teams, ad hoc learning opportunities to acquire new skills and ongoing lack of support from senior staff. These areas emerged without prompting from interviewers. It may be that PRHOs early in their post and low in confidence are worried by the possibility of inadequate support which might come from the unstable teams. However, the reports suggested that it was causing real concern for the interviewees who responded in this way.

It is important that these areas are addressed for the introduction of the Foundation programmes. It is possible that changes in working patterns may mean that those in F1 posts in the future will have to relate to larger teams with a broader range of skills with clear responsibilities but less opportunity for continuity of care. Instability in these larger teams could be even more of a problem. Both PRHOs and more senior doctors will have to learn such new ways of working. The loss of traditional clinical "firms" also needs to be addressed in undergraduate training. Many medical schools retain periods of apprenticeship, teaching traditionally and linking a small number of students to a small clinical team. This allows the students to relate to the staff and build their confidence. The combination and interaction of these teams and the increase in day and outpatient care means that new patterns have already been developed to optimise the students' experience in the hospital environment. Changes in the student experience need to be developed in line with Foundation programmes to be sure that junior doctors are prepared for their roles on graduation.

The greatest satisfaction for PRHOs in this study came from involvement with a clear role and feeling part of the team, and this will be one of the core competencies assessed in Foundation programmes. Mechanisms of working within these teams and handover arrangements need to be clear. While the increased attention on evaluation should lead to clarity of roles with appropriate supervision, the greater emphasis on evaluation could also change the nature of the relationships.

## Conclusion

Overall this study emphasises the enthusiasm of PRHOs for well organised structures with adequate supervision in a supportive multidisciplinary environment. Over the period studied rotas and unstable staffing patterns affected this environment significantly.

## Competing interests

Dr. Mac Cochrane received funding from the Oak Foundation for both studies.

## Authors' contributions

HL developed study design, carried out most interviews, conducted the analyses and wrote the paper. MC supervised study, commented on study design, carried out few interviews, assisted in the data analysis. JR supervised the study, commented on study design, data analysis and contributed in the writing of paper.

## Pre-publication history

The pre-publication history for this paper can be accessed here:



## Supplementary Material

Additional File 1 Microsoft word file (teamwork additional.doc) containing details of the semi-structured interview.
Click here for file
