# Supplementary material for: A qualitative study of the perceptions and experiences of Pre-Registration House Officers on teamwork and support
Source: BMC Med Educ. 2005 Mar 9;5:10. doi: 10.1186/1472-6920-5-10 (PMC1079846; doi:10.1186/1472-6920-5-10)
Supplement: Additional File 1 — Microsoft word file (teamwork additional.doc) containing details of the semi-structured interview. [file 1472-6920-5-10-S1.doc]

**Additional files**

**Semi-structured interview: (PRHOs 2001/2002)**

3 main key topics

Reiterate the aim of the interview (study) and read/tell PRHO the main area you will ask questions about. Remind interviewee that s/he can stop the tape recorder any time. Tell interviewee the approximate length of time the interview might take.

### 1) General perceptions of the first few months and Y5 student rotations

- How has the first month been in your new post as a PRHO?
- What rotation are you in? (how busy have you been)
- How much do you feel part of the ward team now?

### 2) Specific experiences of your PRHO post

 How have your expectations matched the reality of your PRHO post? In the literature the PRHO post is often described as the most stressful period in the medical career.

- Describe a typical day’s work (including ‘intake and on-call duties)
- How many hours do you work with the new working arrangements? (How do you feel about this?)
- How would you describe your relationship with patients since your qualification? (e.g. making a difference)
- Do you feel respected by colleagues and patients? Have you experienced any bullying or public humiliation?

**3) Preparation for the PRHO post**

 Particular the newly developed 5^th^ Year aspires to prepare you well for the PRHO post, however there is some evidence/claim in the medical-educational literature that the undergraduate learning objectives are not always met.

- Have you felt that your core knowledge has been adequate for carrying out your PRHO tasks? (did you feel adequately prepared?)
- Are your clinical skills of history taken and examination appropriate for the job? Are they affected by work pressure?
- Do you feel able to formulate investigations and management strategies for the majority of your patients?
- During the fifth year you completed a number of core skills, which of these did you feel well prepared for in the job and which of them do you find difficult? (list in Y5 handbook/log book)
- Which administrative tasks have you found most difficult? Did you gain any experiences with them during Y5, including IT skills?
- Communication skills had a high profile in Y5, particularly explaining procedures to patients. Have you felt comfortable with your skills and in what situations have you felt are difficulties?
- Have you encountered any ethical dilemmas in your first months as a PRHO?
- How relevant are the SSMs for your new post? (Y5 and previous ones)
- What is your opinion about the final OSCEs?
- How is your perception about the relevance of the ‘campus blocks’ now ?
- Do you feel you are working to your full potential? If not in what way?
- What did you feel was missing from Y5, looking back at your last year in Medical School?
- What professional and personal advice would you give to the next cohort of students for their first weeks as PRHOs?

This is the end of my questions, are there any other issues you would like to talk about which I have not included in the interview.

**End:** debrief student and make own reflective notes how the interview went from your perspective as the interviewer. Check briefly that tape is ‘working’.
